# Does full sternotomy have more significant impact than the cardiopulmonary bypass time in patients of mitral valve surgery?

**DOI:** 10.1186/s13019-018-0719-4

**Published:** 2018-04-14

**Authors:** Zhibing Qiu, Xin Chen, Yueyue Xu, Fuhua Huang, Liqiong Xiao, Ting Yang, Li Yin

**Affiliations:** Department of Thoracic and Cardiovascular Surgery, Nanjing First Hospital, Nanjing Medical University, Changle Rd 68, Nanjing, 210006 Jiangsu People’s Republic of China

**Keywords:** Minimally invasive, Mitral valve surgery, Sternotomy

## Abstract

**Background:**

Over the past decade, minimally invasive mitral valve surgery (MIMVS) has grown in popularity. Less invasive approaches to mitral valve surgery are increasingly used for improved cosmesis. We sought to compare these minimally invasive approaches fairly with conventional full sternotomy approaches by using propensity-matching methods.

**Methods:**

From January 2011 to January 2017, a total of 1120 isolated mitral valve operations were performed at our institution. Data were retrospectively collected on all patients, and a logistic regression model was created to predict selection to a minimally invasive versus conventional sternotomy approach. Propensity scores were then generated based on the regression model and matched pairs created using 1:1 nearest neighbor matching. There were 165 matched pairs in the analysis (sternotomy, *n* = 165;MIMVS, n = 165). Clinical outcomes included bypass and cross-clamp time, length of hospitalization, morbidity, and mortality. Patient details and follow-up outcomes were compared using multivariate, and Kaplan–Meier analyses.

**Results:**

The minimally invasive approach led to slightly longer cardiopulmonary bypass time (99 ± 25 vs 88 ± 17 min, *p* <0.001), and cross-clamp time (65 ± 13 vs 49 ± 11 min, *p*<0.001). Overall, no significant differences existed among major in-hospital complications between groups. There were no differences between the matched groups in 30-day mortality (1.2% vs 0.6%, *p* >0.05). However, Chest tube drainage was lower at 6 and 24 h after a minimally invasive approach (30 ± 5 mL) and 120 ± 20 mL than after conventional sternotomy 175 ± 50 mL and 400 ± 150 mL at these times (*p* < 0.001). Transfusion was less frequent after minimally invasive surgery than after conventional surgery (15.7% vs 40.6%, *p* < 0.001). Patients undergoing minimally invasive surgery spent less time on ventilation support (6.2 ± 1.1 h vs 10.4 ± 2.7, *p* < 0.001). The multivariable regression analysis showed the full sternotomy was an independent risk factor for the propensity-adjusted likelihood of postoperative transfusion, re-exploration for bleeding, and postoperative ventilation support (*p* < 0.05). But the duration of cardiopulmonary bypass time was not an independent risk factor. The mean duration of survival follow-up was 4.4 ± 1.2 years. However, comparison of survival curves between the two groups revealed no significant difference (*P* = 0.203). With regard to freedom from valve-related morbidity, there was no significant difference between groups (P = 0 .574).

**Conclusion:**

Within that portion of the spectrum of mitral valve surgery in which propensity matching was possible, minimally invasive mitral valve surgery has cosmetic, blood product use, and respiratory advantages over conventional surgery, and no apparent detriments. However, minimally invasive mitral valve surgery required a slightly longer cardiopulmonary bypass time and cross-clamp time. Minimally invasive mitral valve surgery represents a safe and effective surgical technique that we believe should be used more routinely in the surgical management of mitral valve disease. MIMVS provides equally durable midterm results as the standard sternotomy approach.

## Background

Right lateral minithoracotomy(RT)has become the standard approach for mitral valve surgery in many centers and is considered to be minimally invasive mitral valve surgery (MIMVS). There is still ongoing debate about the benefits of minimally invasive interventions [1, 2].

Although these benefits, criticisms have been raised as MIMVS is technically more complex, requires a distinct learning curve [3]. Furthermore, the minimally invasive access is technically more demanding and is usually limited to centers with extensive expertise [4, 5]. Patients with left ventricular dysfunction, advanced age, poorer New York Heart Association functional class, larger body mass index, carotid disease, and tricuspid valve regurgitation are more likely to undergo a median sternotomy [6, 7]. Therefore, basic characteristics of patients selected for median sternotomy and minimally invasive access are usually not comparable, and prospective randomized trials are not available. Since 2011, we started our MIMVS program and after few years, RT approach has become the standard approach for the treatment of isolated mitral valve disease. The aim of our study is to report early and mid-term outcomes of consecutive patients who had undergone mitral valve surgery using RT during a 6-year period.

## Methods

### Patient selection and data collection

From January 2011 to January 2017, 1120 patients underwent primary isolated mitral valve surgery. Minimally invasive surgery was performed in 283 (25.3%) patients, and conventional full sternotomy was performed in 837 (74.7%) patients. The present study was a retrospective, observational review. The institutional review board approved the study and waived patient consent. Patient selection was predicated by surgeon preference. The patient demographics, medical history, and operative and in-hospital outcomes were collected at each patient’s admission and at each consecutive follow-up visit. Mid-term mortality was documented, with a review of the data collected from routine clinic follow-up visits, in addition to a query of the Social Security Death Index. Propensity scores were then generated based on the regression model and matched pairs created using 1:1 nearest neighbor matching. There were 165 matched pairs in the analysis for a total sample size of 1120(sternotomy group, *n* = 165; minimally invasive group, n = 165).

### Exclusion criteria

Patients with concomitant coronary artery bypass graft surgery, aortic or tricuspid valve procedures, and surgical ablation of atrial fibrillation were excluded from the study. Studies including mainly redo surgical procedures were excluded. The cause of the mitral valve disease was not taken into consideration for inclusion or exclusion of the studies.

### Definitions

Hospital mortality included all deaths within 30 days of operation irrespective of where the death occurred and all deaths in hospital after 30 days among patients who had not been discharged after the index operation. The diagnosis of stroke was also confirmed by computed tomography or magnetic resonance imaging whenever possible and documented by staff neurologists. Renal complications included acute renal failure, defined as the requirement for hemodialysis or an increased creatinine level (> 200 mmol/L). A diagnosis of postoperative myocardial infarction was based on the presence of new Q waves greater than 0.04 milliseconds and/or a reduction in R waves greater than 25% in at least 2 contiguous leads on an electrocardiogram. Pulmonary complications included chest infection, ventilation failure, reintubation, and tracheostomy.

### Surgical technique

Briefly, MIMVS by a way of right anterior thoracotomy was performed through a 5–7 cm skin incision placed at 4th intercostal space. After incision a soft tissue retractor is inserted and the intercostal space is gently spread with a retractor. Two trocars are inserted in the thorax to allow positioning of a ventricular vent, CO_2_ insufflator, camera device and pericardial stay sutures. Cardiopulmonary bypass was initiated by way of the femoral artery and vein cannulation through a small transverse incision in the groin,and direct transthoracic aortic clamping. QuickDraw Venous Cannula single stage (Edwards Lifesciences) were inserted through the femoral vein into the right atrium and the correct position was achieved with the Seldinger technique under transesophageal echocardiographic guidance. Direct ascending aorta cannulation is performed under direct vision. After vacuum-assisted cardiopulmonary bypass (− 40 to − 60 mmHg) was established, the patients were cooled to 34 C°. The lungs must be deflated before aortic cannulation. The aorta was crossclamped using the Chitwood aortic clamp (Cardiomedical GmbH, Langenhagen, Germany) directly through the thoracotomy incision, and antegrade cold crystalloid blood cardioplegia is delivered directly into the ascending aorta by a needle vent catheter. The mitral valve is approached with a traditional left paraseptal atriotomy and exposed using a specially designed atrial retractor held by a mechanical harm inserted through a right parasternal port. Mitral valve procedures were performed under a combination of direct vision and thoracoscopic assistance. All patients received an accurate intraoperative transoesophageal echocardiogram before and after weaning from cardiopulmonary bypass machine.

### Full sternotomy

Standard operative technique was a median sternotomy and cardiopulmonary bypass using aortic and bicaval cannulation. Cardiac arrest was induced by the instillation of antegrade cardioplegia. The operative strategy was individualized but aimed towards curative resection.

### Follow-up

Complete follow-up could be achieved in 92.1% (mean follow-up 4.4 ± 1.2 years). Information was collected from patient’s follow-up visits, telephone interviews with the patient or the referring physician, and mailed questionnaires. Postoperative complications were analyzed according to the “Guidelines for Reporting Morbidity and Mortality after Cardiac Valvular Operations,” approved by The Society of Thoracic Surgeons.

### Statistical analysis

Continuous data were expressed as mean ± standard deviation or median with the interquartile range and categorical data as percentages. Cumulative survival was evaluated with the Kaplan–Meier method. All reported *P* values are two-sided, and P values of < 0.05 were considered to indicate statistical significance. All statistical analyses were performed with SPSS 22.0 (SPSS, Inc., Chicago, IL, USA). All statistical analyses were performed with the assistance of a departmental statistician.

A propensity score, indicating the predicted probability of receiving MIMVS treatment, was then calculated by the use of a non-parsimonious multiple logistic regression analysis from the logistic equation for each patient. Finally, we used the propensity score to match MIMVS to Sternotomy patients (1:1 match). Results are reported as percentage and odds ratios (ORs) and 95% confidence intervals. The propensity score included the following variables: age, body surface area, the ratio of rheumatic cause, preoperative ejection fraction (EF), sex, CPB time, New York Heart Association (NYHA) functional class (I, II versus III, IV), diabetes mellitus and hypertension.

Multiple variable models were constructed to determine independent factors influencing the following outcomes: postoperative blood transfusion, reoperation for early postoperative hemorrhage, length of postoperative ventilation support, and length of hospital stay after surgery.

## Results

### Patient characteristics

Table 1 compares patient demographics between the two surgical approaches. Although the patient ages and pulmonary artery pressures were similar, the preoperative New York Heart Association (NYHA) functional classification was worse in the group undergoing the sternotomy approach (2.6 ± 0.6 vs 2.1 ± 0.5, *P* < .001). After propensity score matching there were 165 matched pairs of patients (Table 1). Once matched, there were no longer significant differences among major baseline characteristics between groups, including gender, age, the ratio of rheumatic cause, cerebral infarction, pre-operative left ventricular ejection function, and so on.

**Table 1:** Characteristics of Unmatched and Propensity Matched Patients

Characteristics	Unmatched Patients(*n* = 1120)			Matched Patients(*n* = 165)		
	Sternotomy(*n* = 837)	MIMVS(*n* = 283)	*p* Value	Sternotomy(n = 165)	MIMVS(n = 165)	*p* Value
Age (years)	57.5 ± 8.3	46.7 ± 7.2	0.000	52.6 ± 7.0	51.5 ± 6.8	0.1486
Sex (female)	485(57.9%)	108(38.2%)	0.000	56(33.9%)	58(35.2%)	0.817
Body mass index	25.2 ± 2.2	24.5 ± 1.3	0.000	25.6 ± 2.0	25.5 ± 2.0	0.6500
Rheumatic valvular disease (n)	468(55.9%)	162(57.2%)	0.384	94(57.0%)	96(58.2%)	0.487
Hypertension	302(36.1%)	60(21.2%)	0.000	36(21.8%)	35(21.2%)	0.893
Diabetes mellitus	143(17.1%)	56(19.8%)	0.304	30(18.1%)	31(18.8%)	0.887
Preoperative creatinine (mg/dL)	0.72 ± 0.19	0.77 ± 0.10	0.000	0.74 ± 0.13	0.75 ± 0.12	0.4683
LVEF, %	56.7 ± 6.9	61.6 ± 4.7	0.000	59.2 ± 4.2	59.5 ± 4.0	0.5069
Current congestive heart failure	259(30.9%)	51(18.0%)	0.000	38(23.0%)	36(21.8%)	0.792
History of AF	378(45.2%)	85(30.0%)	0.000	60(36.4%)	58(35.2%)	0.818
COPD	134(16.0%)	26(9.2%)	0.005	20(12.1%)	18(10.9%)	0.730
Cerebrovascular disease	76(9.1%)	14(4.9%)	0.027	11(6.7%)	10(6.1%)	0.822
preoperative NYHA functional class	2.6 ± 0.6	2.1 ± 0.5	0.0000	2.3 ± 0.5	2.3 ± 0.4	1.0000
NYHA III-IV functional class, n (%)	242(28.9%)	43(15.2%)	0.000	34(20.6%)	32(19.4%)	0.783
Pulmomary Hypertension (≥60 mmHg)	252(30.1%)	78(27.6%)	0.417	47(28.5%)	46(27.9%)	0.903
EuroSCORE I	6.5 ± 1.0	4.8 ± 0.8	0.000	5.9 ± 0.9	5.8 ± 0.8	0.287

AF = atrial fibrillation; COPD = chronic obstructive pulmonary disease; NYHA = New York Heart Association; EuroSCORE I = European System for Cardiac Operative Risk Evaluation, version I

### Operative and postoperative data

Several differences in operative and postoperative variables were identified on univariate analysis (Table 2). Minimally invasive patients had significantly longer cross-clamp time (65 ± 13 versus 49 ± 11 min, *p*<0.001) and bypass time (99 ± 25 versus 88 ± 17 min, *p*<0.001). No aortic dissections or injury occurred in either patient group. Patients undergoing minimally invasive surgery spent less time on ventilation support (6.2 ± 1.1 h vs 10.4 ± 2.7, *p* < 0.001).

**Table 2:** Perioperative Data of matched pairs

Variable	Sternotomy (n = 165)	MIMVS(n = 165)	*p* Value
Cross-clamp time, minutes	49 ± 11	65 ± 13	0.0000
Bypass time, minutes	88 ± 17	99 ± 25	0.0000
Mitral valve repair	52(31.5%)	50(30.3%)	0.657
Ventilation time, hours	10.4 ± 2.7	6.2 ± 1.1	0.0000
New onset of AF, n	51(30.9%)	43(26.1%)	0.329
Stroke, n	2(1.2%)	3(1.8%)	1.000
Blood transfusion, n	67(40.6%)	26(15.7%)	0.000
Reoperation for bleeding, n	4(2.4%)	1(0.6%)	0.367
Drainage Postoperative 6 h(ml)	175 ± 50	30 ± 5	0.0000
Drainage Postoperative 24 h(ml)	400 ± 150	120 ± 20	0.0000
Deep wound infection, n	6(3.6%)	0	0.039
Prolonged ventilation(>24 h)	10(6.1%)	3(1.8%)	0.048
Intensive care unit stay (h)	30.6 ± 19.5	24.3 ± 9.7	0.0002
Postoperative length of stay(days)	10.5 ± 2.0	8.0 ± 1.0	0.0000
In-hospital mortality	2(1.2%)	1(0.6%)	1.000

AF = atrial fibrillation

Reoperation for bleeding was similar in matched groups (0.6%in MIMVR group vs 2.4%in the conventional sternotomy group, (*P* = 0.367; Table 2). However, Chest tube drainage was lower at 6 and 24 h after a minimally invasive approach (30 ± 5 mL) and 120 ± 20 mL than after conventional sternotomy 175 ± 50 mL and 400 ± 150 mL at these times (*p* < 0.001). Transfusion was less frequent after minimally invasive surgery than after conventional surgery (15.7% vs 40.6%, *p*<0.001). In-hospital complications are summarized in Table 2. Overall, no significant differences existed among major in-hospital complications between groups. There were 6 sternal wound infections (0.79%) among patients in the ST group. There was no significant difference in survival at 30 days between groups (*p* = 1.0). The hospital mortality was 1.2% for the sternotomy and 0.6% for the minimally invasive approach. Permanent neurologic perioperative events occurred in 1.2% of patients undergoing sternotomy and 1.8% of the patients undergoing the minimally invasive approach.

The multivariable regression analysis showed the full sternotomy was an independent risk factor for the propensity-adjusted likelihood of postoperative transfusion, re-exploration for bleeding, and postoperative ventilatory support (*p* < 0.05). Another independent predictor of length of hospital stay was ejection fraction (*P* = 0.006; Table 3). But the duration of cardiopulmonary bypass time was not an independent risk factor.

**Table 3:** Multivariate Analysis

Variable	Odds Ratio	95%CI	*p* Value
Transfusion			
Sternotomy	1.01	1.00–1.02	0.003
Propensity	1.39	1.02–1.09	0.039
CPB time	0.97	0.83–1.14	0.82
Reoperation for hemorrhage			
Sternotomy	2.02	1.24–3.27	0.005
CPB time	4.775	0.27–85.29	0.288
Propensity	1.65	1.23–2.18	0.001
Ventilator			
Sternotomy	1.92	1.13–3.16	0.012
CPB time	1.495	0.09–24.57	0.778
Propensity	2.45	1.46–4.18	0.008
Hospital stay			
Sternotomy	0.86	0.55–1.38	0.53
Propensity	1.07	0.96–1.17	0.23
CPB time	2.02	0.76–5.1	0.18
Ejection fraction	0.98	0.29–1.71	0.006

CPB = cardiopulmonary bypass;

### Follow-up

**Fig. 1:**
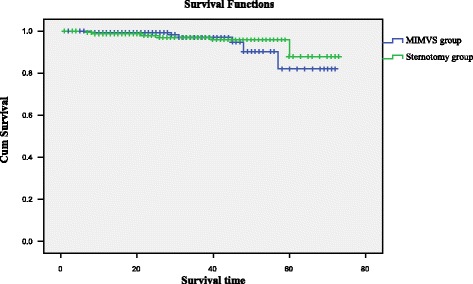
Kaplan-Meier analysis of long-term survival.(Blue line = minimally invasive; green line = sternotomy

**Fig. 2:**
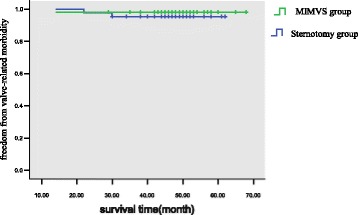
Kaplan-Meier estimate of freedom from mitral valve-related morbidity of patients with either a right minithoracotomy (green line) or a median sternotomy (blue line)

Late death occurred in 10 patients (2 cardiac-related deaths, and 8 non–cardiac related deaths).The mean New York Heart Association class at follow-up was 1.5 ± 0.6. There was 1 late re-intervention at 1.1 year for mitral prosthesis endocarditis (Table 4). Survival at 1, 3, and 5 years was 98.1% ± 0.9%, 93.5% ± 2.7%, and 91.7% ± 3.8%, respectively Among matched patients, survival at 1, 3, and 5 years was 99.3 ± 0.7%, 97.3 ± 1.5%, and 92.1 ± 2.6% after MIMVS surgery and 98.7 ± 1.0%, 96.2 ± 1.7%, and 92.5 ± 2.7% after conventional sternotomy. However, comparison of survival curves between the two groups revealed no significant difference (*P* = 0.203, Fig. 1). With regard to freedom from valve-related morbidity, there was no significant difference between groups (P = 0 .574, Fig. 2).

**Table 4:** Follow-up Results of Propensity Matched Patients

Complications	Sternotomy (*n* = 152)	MIMVS (n = 152)	χ^2^值	*P* Value
Valve-related	5(3.3%)	4(2.6%)	0.000	1.000
Bleeding event	1(0.7%)	2(1.3%)	0.000	1.000
Thromboembolism	1(0.7%)	1(0.7%)	0.000	1.000
PVE	1(0.7%)	1(0.7%)	0.000	1.000
Valve deterioration	0	0	/	1.000
perivalvular leak	1(0.7%)	0	/	1.000
Reoperation	1(0.7%)	0	/	1.000
Cardiac death	1(0.7%)	1(0.7%)	0.000	1.000
Heart failure	1(0.7%)	0	/	1.000
Arrhythmia	0	1(0.7%)	/	1.000
Non-cardiac death	4(2.6%)	4(2.6%)	0.000	1.000
Malignancy	3(1.9%)	2(1.3%)	0.000	1.000
Other	1(0.7%)	2(1.3%)	0.000	1.000
Late mortality	5(3.3%)	5(3.3%)	0.000	1.000

PVE = prosthetic valve endocarditis

## Discussion

Over the past decade, the field of MIMVS cardiothoracic surgery has seen rapid growth, and Mitral valve surgery has been particularly amenable to minimal access approaches, and the described benefits in the literature include improved patient satisfaction, improved cosmesis, decreased length of hospitalization, and decreased overall resource utilization [8–10]. Unfortunately, previous studies have been limited either by descriptions of MIMVS outcomes with no reference group for comparison, or a sternotomy reference group with significant differences in baseline risk [11–13]. To address these challenges, we used a propensity-matched comparison technique to simulate outcomes after pseudo randomization of patients to Sternotomy versus MIMVS approach for mitral valve surgery.

Bleeding was minimized, but this did not translate into less frequent surgical re-exploration or lower transfusion requirements. However, previous series [14–16], including our propensity- matched comparison of mitral valve procedures with a larger series of patients (*n* = 165) receiving isolated, less invasive valve surgery, have shown benefits compared with full sternotomy. The reasons for reduced bleeding are most likely associated with a smaller incision and less dissection of tissue and thus a smaller wound surface. Diffuse bleeding should therefore be less. And serratus anterior muscle was blunt dissected and only the intercostal muscle should be cut off, without disruption of the integrity of the thorax, so that the surgery was with little trauma, better for postoperative recovery. Using propensity adjustment, we were able to demonstrate an advantage of a MIMVS approach in diminishing postoperative blood transfusion, reoperation for hemorrhage, or postoperative length of stay.

Not surprisingly, sternal wound infections were significantly decreased with mini-MVS versus conventional MVS (0% vs 3.6%). This is a result that is consistent with expectations, as the mini-MVS approach was by definition via a thoracotomy rather than via sternotomy, whereas the conv-MVS approach was by definition only via median sternotomy in this meta-analysis [17, 18].

However, despite the potential benefits of mini-MVS and the results of our study to suggest the mid-term durability to be maintained using these techniques, mini-MVS has potential drawbacks that still need to be addressed. An increased risk of stroke, aortic dissection, and groin complications and increased crossclamp and cardiopulmonary times have all been mentioned as being of greater risk when performing mini-MVS versus conventional techniques [19, 20]. In our particular series, such complications have remained low. The current study demonstrates that despite significantly longer cross-clamp and bypass times, the early outcomes of MIMVS are similar to those of an open approach through median sternotomy.

Similar results were described by the Society of Thoracic Surgeons of the adult cardiac surgery database as well as by several meta-analyses confirming the main points of the aforementioned consensus statement [5, 6, 21]. Our results are in line with the current literature; however, despite these excellent outcomes, many criticism still remain regarding MIMVS as it is technically more complex, requires a distinct learning curve (prolonged cross-clamp and cardiopulmonary bypass times). Finally, for many surgeons, the decision to utilize MIMVS is more related to the cosmetic results than better clinical outcomes, because no large randomized trial has been performed.

Despite these differences, in both the current series and others [22, 23], early mortality was not increased in the port group. With regard to survival, there was no difference in 1-year or 3-year mortality between groups, and both MIMVS and ST patients had excellent short-term survival. Mid-term survival was also similar between groups, with both groups achieving survival rates above 92% at 5 years after surgery. Thus, in our series, a minimal access approach for mitral valve surgery does not appear to compromise morbidity or mortality when compared with matched ST controls.

### Limitations

The main limitation of this study is its retrospective nature. This is also a single-institution study, which limits its generalizability. Within this single-institution experience, we acknowledge that selection bias cannot be completely reversed by propensity-based methods and in this study cannot completely overcome distinct surgeon preferences. Whether a larger series of patients with more power would have shown more benefits is unknown.

## Conclusions

In this series, we demonstrate through propensity matching that a MIMVS approach for mitral valve surgery is associated with slightly increased CPB times and cross-clamp times when compared with an ST approach. Moreover, we show that a MIMVS approach is associated with equivalent rates of morbidity and mortality with the benefit of decreased postoperative blood transfusion, reoperation for hemorrhage, or postoperative length of stay, and reduced assisted ventilation duration. In addition to improved cosmetic results, MIMVS provides equally durable midterm results as the standard sternotomy approach. In conclusion, MI mitral valve surgery represents a safe and effective surgical technique that we believe should be used more routinely in the surgical management of mitral valve disease. However, widespread acceptance of this technique requires further advances in technique and proof of benefits in propensity-matched patients, because a randomized trial appears unlikely.

## Abbreviations

CPB: Cardiopulmonary bypass
EF: Ejection fraction
MIMVS: Minimally invasive mitral valve surgery
MVS: Mitral valve surgery
NYHA: New York Heart Association
RT: Right lateral minithoracotomy
ST: Sternotomy

## References

[1] Schmitto JD, Mokashi SA, Cohn LH (2010). Minimally-invasive valve surgery. J Am Coll Cardiol.

[2] Goldstone AB, Atluri P, Szeto WY, Trubelja A, Howard JL, MacArthur Jr JW (2013). Minimally invasive approach provides at least equivalent results for surgical correction of mitral regurgitation: a propensity-matched comparison. J Thorac Cardiovasc Surg.

[3] Holzhey DM, Seeburger J, Misfeld M, Borger MA, Mohr FW (2013). Learning minimally invasive mitral valve surgery: a cumulative sum sequential probability analysis of 3895 operations from a single high-volume center. Circulation.

[4] Pope NH, Ailawadi G (2014). Minimally invasive valve surgery. J Cardiovasc Transl Res.

[5] Modi P, Hassan A, Chitwood WR (2008). Minimally invasive mitral valve surgery: a systematic review and meta-analysis. Eur J Cardiothorac Surg.

[6] Falk V, Cheng DC, Martin J, Diegeler A, Folliguet TA, Nifong LW (2011). Minimally invasive versus open mitral valve surgery: a consensus statement of the international society of minimally invasive coronary surgery (ISMICS) 2010. Innovations (Phila).

[7] Sündermann SH, Czerny M, Falk V (2015). Open vs. minimally invasive mitral valve surgery: surgical technique, indications and results. Cardiovasc Eng Technol.

[8] Cosgrove DM, Sabik JF, Navia JL (1998). Minimally invasive valve operations. Ann Thorac Surg.

[9] Gilmanov D, Bevilacqua S, Murzi M, Cerillo AG, Gasbarri T, Kallushi E (2013). Minimally invasive and conventional aortic valve replacement: a propensity score analysis. Ann Thorac Surg.

[10] Cohn LH, Byrne JG (2013). Minimally invasive mitral valve surgery: current status. Tex Heart Inst J.

[11] Cheng DCH, Martin J, Lal A, Diegeler A, Folliguet TA, Nifong LW (2011). Minimally invasive versus conventional open mitral valve surgery. Innovations.

[12] Cao C, Gupta S, Chandrakumar D, Nienaber TA, Indraratna P, Ang SC (2013). A meta-analysis of minimally invasive versus conventional mitral valve repair for patients with degenerative mitral disease. Ann Cardiothorac Surg.

[13] Grossi EA, LaPietra A, Ribakove GH (2001). Minimally invasive versus sternotomy approaches for mitral reconstruction: comparison of intermediate-term results. J Thorac Cardiovasc Surg.

[14] Svensson LG, Atik FA, Cosgrove DM, Blackstone EH, Rajeswaran J, Krishnaswamy G (2010). Minimally invasive versus conventional mitral valve surgery: a propensity-matched comparison. J Thorac Cardiovasc Surg.

[15] Ryan WH, Brinkman WT, Dewey TM, Mack MJ, Prince SL, Herbert MA (2010). Mitral valve surgery: comparison of outcomes in matched sternotomy and port access groups. J Heart Valve Dis.

[16] Holzhey DM, Shi W, Borger MA (2011). Minimally invasive versus sternotomy approach for mitral valve surgery in patients greater than70 years old: a propensity-matched comparison. Ann Thorac Surg.

[17] Banbury MK, White JA, Blackstone EH, Cosgrove DM (2003). Vacuum-assisted venous return reduces blood usage. J Thorac Cardiovasc Surg.

[18] Melnitchouk SI, Dal-Bianco JP, Borger MA (2015). Minimally invasive mitral valve surgery via mini-thoracotomy: current update. Curr Treat Options Cardio Med.

[19] Iribarne A, Russo MJ, Easterwood R, Hong KN, Yang J, Cheema FH (2010). Minimally invasive versus sternotomy approach for mitral valve surgery: a propensity analysis. Ann Thorac Surg.

[20] Lange R, Voss B, Kehl V, Mazzitelli D, Tassani-Prell P, Günther T (2017). Right Minithoracotomy versus full sternotomy for mitral valve repair: propensity matched comparison. Ann Thorac Surg.

[21] Schmitto JD, Mokashi SA, Cohn LH (2011). Past, present, and future of minimally invasive mitral valve surgery. J Heart Valve Dis..

[22] Akowuah E, Burdett C, Khan K, Goodwin A, Lage IB, El-Saegh M (2015). Early and Late Outcomes After Minimally Invasive Mitral Valve Repair Surgery. J Heart Valve Dis..

[23] Gammie JS, Bartlett ST, Griffith BP (2009). Small-incision mitral valve repair: safe, durable, and approaching perfection. Ann Surg.

